# Incomplete superficial palmar arch and bilateral persistent median artery

**DOI:** 10.1016/j.ijscr.2019.04.035

**Published:** 2019-04-30

**Authors:** Chirag Buch, Candice M. Devora, Linda Y. Johnson, Omid B. Rahimi, Rekha Kar

**Affiliations:** a Department of Cell Systems and Anatomy, University of Texas Health at San Antonio, San Antonio, TX 78229, USA; b Long School of Medicine, University of Texas Health at San Antonio, San Antonio, TX 78229, USA

**Keywords:** Incomplete palmar arch, Persistent median artery, Vascular anomaly

## Abstract

**Introduction:**

The superficial palmar arch (SPA) is a major source of blood supply to much of the hand and is conventionally formed by the anastomosis of the ulnar artery with the superficial branch of the radial artery. The SPA has been classified into complete or incomplete based on the presence or absence of anastomosis between the arteries contributing to the formation of this palmar arch.

**Case report:**

Reported here is a unilateral presentation of incomplete superficial palmar arch. The ulnar artery (UA) gave off one proper palmar digital artery, which supplied the ulnar side of the little finger, and two common palmar digital arteries, which supplied the little, ring, and the middle finger. The superficial palmar branch of the radial artery gave off a proper palmar digital artery to the thumb, and two common palmar digital arteries, which supplied the thumb, index, and middle fingers. Apart from the presence of the incomplete SPA, persistent median arteries were also observed bilaterally in this cadaver.

**Discussion:**

The prevalence of incomplete SPA has been reported to vary between 3.6–54.76%. To the best of our knowledge, this is the first case report describing an incomplete palmar arch and bilateral persistent median artery in a cadaver.

**Conclusion:**

Patients should be screened for the presence of complete or incomplete SPA before harvesting the radial artery either for myocardial revascularization or for radial artery forearm flap to prevent ischemic complications in the hand.

## Highlights

• Possible anatomical variations of the vascular supply for the hand.
• Screen patients for the presence of persistent median artery.

## 1 Introduction

In most cases, the superficial palmar arch (SPA) is formed by superficial branches of both the ulnar artery and the radial artery. The SPA has been classified into complete or incomplete based on the presence or absence of anastomosis between the arteries contributing to the formation of this palmar arch [1]. The SPA has been reported to be highly variable with regard to its origin and branching pattern in the hand. Several reports have established that the complete SPA can be Radio-ulnar, ulnar, median-ulnar, or Radio-median-ulnar type [[2], [3], [4], [5]]. In the incomplete SPA, the ulnar artery fails to anastomose with the radial and/or the median artery and thus, fails to reach the index finger and the thumb. Coleman et al. [2] defined four main types of incomplete superficial palmar arches: type A, B, C, and D. Type A, observed in 3.6% of cases, where superficial branches of both the ulnar and the radial artery reach the hand, but fail to anastomose with each other and supply the fingers and the palm independent of each other. In type B (13.4%), the ulnar artery forms the SPA, however, it does not supply the index finger and the thumb. Type C (3.8%) receives contributions from both the ulnar and the median arteries, which do not anastomose with each other. In type D (1.1%), the radial, the ulnar and the median artery provide blood supply to the palm and the fingers and these arteries fail to anastomose with each other.

An incomplete SPA can present with substantial clinical implications if a patient is not screened for such anatomical variation during routine or surgical procedures, such as arterial blood sampling, reconstructive hand procedures, artery grafting for cardiac bypass surgery and various microvascular surgeries. This report describes an incomplete SPA in a cadaver. In addition to the presence of an incomplete SPA unilaterally, a persistent median artery was also observed in this cadaver. The median artery provides arterial supply for the forearm and the hand during embryonic development and usually regresses prior to the 8^th^ week of gestation after the development of the radial and the ulnar arteries. However, the median artery may persist in adults and such persistence can lead to many complications involving median nerve compression e.g. carpal tunnel syndrome, anterior interosseous nerve syndrome, and pronator teres syndrome [6]. The reported prevalence of a persistent median artery is highly variable ranging from 1.1 to 16.1%) [7]. This work has been reported in line with the SCARE criteria [12].

## 2 Case report

**Fig. 1:**
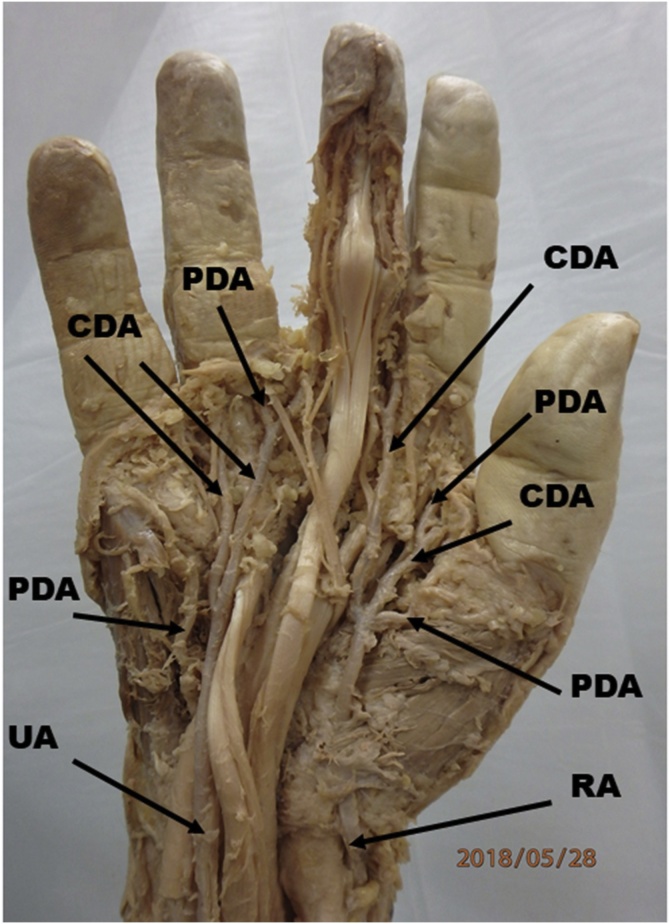
Photograph showing the incomplete superficial palmar arch with both the superficial branches of the ulnar artery (UA) and the radial artery (RA) entering the hand. Both the UA and the RA gave off one proper palmar digital artery (PDA) and 2 common palmar digital arteries (CDA).

**Fig. 2:**
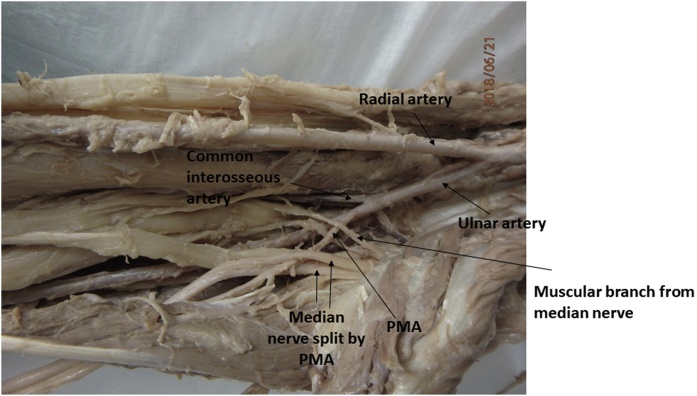
Photograph showing the persistent median artery (PMA) taking origin from the ulnar artery and dividing the median nerve on its course in the forearm.

An incomplete SPA was observed during a routine dissection of the hand for second year medical student education in the right hand of a cadaver who died due to cardiac arrest. The incomplete superficial palmar arch was visualized between the palmar aponeurosis and the long flexor digitorum tendons on the right hand. The superficial branch of the radial artery entered the hand aberrantly deep to the abductor pollicis brevis muscle and gave a proper digital artery to the thumb and two common digital arteries. One of these common digital arteries gave two proper digital arteries which supplied the thumb and the index finger and the other common digital artery gave two proper digital arteries supplying the ulnar side of the index finger and the radial side of the middle finger. The superficial branch of the ulnar artery in the hand gave rise to a proper palmar digital artery to the ulnar side of the little finger. It also gave off two common palmar digital arteries. One of these common palmar digital artery supplied the radial side of the little finger and the ulnar side of the ring finger and the other common palmar digital artery supplied the radial side of the ring finger and the ulnar side of the middle finger (Fig. 1). This cadaver also had bilateral persistent median arteries (PMA) which originated from the ulnar artery and travelled along with the median nerve deep to the pronator teres and the flexor digitorum superficialis muscles. The PMA terminated within the carpal tunnel and did not contribute to the palmar arch and failed to give rise to the palmar digital arteries (Fig. 2).

## 3 Discussion

The prevalence of incomplete SPA has been reported to vary between 3.6–54.76% [[2], [3], [4], [5],[8], [9], [10]]. This discrepancy in the percentage of population exhibiting incomplete palmar arches can at least be partly attributed to the limited number of cadavers included in some studies and/or racial differences.

Based on Coleman’s classification [2], this case report fits in the category of type A incomplete arch, a very rare variation of the superficial palmar arch. Kaplanoglu et al. classified incomplete arches into 3 different categories (E–G) [11]. Type E is where the superficial branch of radial supplied the lateral 2^1/2^ digits and the superficial branch of the ulnar artery supplied the medial 2^1/2^ digits. Type F was similar to type B of Coleman’s classification where only the ulnar artery formed the incomplete arch. In type G, the ulnar artery moves towards the median plane, gives origin to two common digital arteries which further subdivides into two and supplies the 1^st^, 2^nd^ and half of 3^rd^ digits. The present case report fits into the type E of Kaplanoglu’s classification as the superficial branches of ulnar and radial artery supplied 2^1/2^ digits each.

This cadaver also had bilateral persistent median arteries that originated from the ulnar artery. Even though the median artery coursed along with the median nerve and entered the carpal tunnel, it did not give rise to any common digital arteries. Thus, it failed to provide blood supply to any of the digits.

To the best of our knowledge, this is the first case report describing an incomplete palmar arch and bilateral persistent median artery in a cadaver. This report emphasizes the need for clinicians to gain comprehensive understanding of possible anatomical variations of the vascular supply for the hand prior to performing any surgical procedure involving the arteries of the forearm and the hand.

## 4 Conclusion

Since SPA is a main source of blood supply to the hand, it is crucial for clinicians performing reconstructive hand surgeries to know the possible anatomical variations in its connections and branching patterns. Furthermore, patients should be screened for the presence of complete or incomplete SPA using either the Allen’s test or the Doppler flow meter before harvesting the radial artery either for myocardial revascularization or for radial artery forearm flap to prevent ischemic complications in the hand.

## Conflicts of interest

None.

## Sources of funding

None.

## Ethical approval

This was a cadaveric study where donors had submitted their bodies to the Willed body donation program at UT health San Antonio.

## Consent

Body donors used in this study are willed to the Human Anatomy Program at the UT Health San Antonio. UT Health San Antonio is a member institution of the State Anatomical Board of Texas. All donations to the program are registered and cared for under the statutes and guidelines of the State of Texas.

## Author’s contribution

CB and CMD collected, analyzed data and wrote paper, LYJ, OBR provided intellectual inputs on the variation, reviewed and edited the manuscript and RK conceptualized, designed and wrote the paper.

## Registration of research studies

N/A.

## Guarantor

Rekha Kar.

## Provenance and peer review

Not commissioned, externally peer-reviewed.
